# Supplementary material for: A Significant Increasing Risk Association between Cigarette Smoking and XPA and XPC Genes Polymorphisms
Source: Genes (Basel). 2023 Jun 27;14(7):1349. doi: 10.3390/genes14071349 (PMC10379612; doi:10.3390/genes14071349)
Supplement: Supplementary file 1 [file genes-14-01349-s001.zip › genes-2261163-supplementary.pdf]

Table S1. Data for XPA genes SNPs.

Genotyped SNPs	rs10817938	rs1800975	rs3176751	rs3176752
Chromosome		9		
Location	Promoter in −2718 bp from the transcription start site	Intron variant	Intron variant	Intron variant
Chr Pos (Genome Build 36.3)	97700127	97697296	97675236	97675205
Base change	T /C	T/ C	C/G	G/T

Table S2. Data for XPC genes SNPs.

Genotyped SNPs	rs2607775	rs2228000	rs2228001	rs1870134
Chromosome			3	
Location	Intron variant	Exon 9 (Ala499Val)	Exon 16 (Gln939Lys)	Intron variant
Chr Pos (Genome Build 36.3)	14178595	14158387	14145949	14178523
Base change	G/C	C/T	A/C	G/C
